# Is There a Standard Adjuvant Therapy for Resected Pancreatic Cancer?

**DOI:** 10.3390/cancers11101547

**Published:** 2019-10-12

**Authors:** Elisabetta Fenocchio, Roberto Filippi, Pasquale Lombardi, Virginia Quarà, Michela Milanesio, Giacomo Aimar, Francesco Leone, Massimo Aglietta

**Affiliations:** 1 Multidisciplinary Outpatient Oncology Clinic, Candiolo Cancer Institute, FPO-IRCCS, Strada Provinciale 142, km 3.95, 10060 Candiolo (TO), Italy; 2 Department of Oncology, University of Turin Medical School, Strada Provinciale 142, km 3.95, 10060 Candiolo (TO), Italy; roberto.filippi@ircc.it (R.F.); pasquale.lombardi@ircc.it (P.L.); virginia.quara@ircc.it (V.Q.); michela.milanesio@ircc.it (M.M.); giacomo.aimar@ircc.it (G.A.); massimo.aglietta@unito.it (M.A.); 3 Division of Medical Oncology, Candiolo Cancer Institute, FPO-IRCCS, Strada Provinciale 142, km 3.95, 10060 Candiolo (TO), Italy; francesco.leone@unito.it; 4 Department of Oncology, Azienda Sanitaria Locale di Biella, 13875 Ponderano (BI), Italy

**Keywords:** adjuvant chemotherapy, adjuvant chemoradiation, pancreatic ductal carcinoma, capecitabine, gemcitabine, mFOLFIRINOX

## Abstract

Surgical resection remains the only treatment that offers a potential chance of long-term survival. Unfortunately, about 80% of patients treated with curative intent will develop recurrence. Since 2001, adjuvant therapy with gemcitabine or 5-fluorouracyle was recommended. This approach allows a median overall survival (OS) of around 23 months, and 5-year survival of 22%. In recent years, two phase-3 trials investigating new chemotherapy regimens resulted in considerably improved survival times. The doublet gemcitabine–capecitabine has shown improvement in OS from 25.5 to 28 months (*p* = 0.032) compared to gemcitabine, in the ESPAC-4 trial. Later, preliminary results of PRODIGE 24 trial presented at the 2018 ASCO meeting showed a superiority of a combination chemotherapy regimen with fluorouracil, leucovorin, irinotecan, and oxaliplatin (mFOLFIRINOX) when compared to gemcitabine alone, both in terms of median disease-free survival (21.6 vs. 12.8 months, *p* < 0.0001) and OS (54.4 vs. 35 months, *p* = 0.003). Contrary to chemotherapy, the role of adjuvant radiotherapy is still controversial, even in the case of R1 surgery. A randomized trial exploring the role of chemoradiotherapy in this setting is now ongoing in the US (RTOG-0848). Overall, the management of localized pancreatic adenocarcinoma is evolving. In this review, we summarize the current status and the most up-to-date developments in adjuvant treatment.

## 1. Introduction

Pancreatic ductal adenocarcinoma (PDAC) is characterized by dismal prognosis, with the lowest survival rate of all the common malignancies worldwide, with an estimated 5-year (y) survival rate of <5% [1].

Responsible for over 95,000 deaths every year in Europe, PDAC is the third-leading cause of cancer-related death, behind lung and colorectal cancer, and it is expected to become the second-leading cause of cancer death by 2020 [2].

Despite recent advances in immunotherapy and perioperative therapy, resulting in more successful resections and targeted treatments, PDAC mortality has steadily risen over the past few decades [3,4,5].

For early-stage PDAC, surgical resection with negative margins represents the treatment option with the best chances of long-term survival, but outcomes remain poor. Among the 20% of patients (pts) diagnosed with a localized PDAC, the disease recurs in up to 80% within 2 years [6,7,8].

Therefore, an adjuvant treatment has been advocated to increase the percentage of pts with long-term survival after surgery. Multiple clinical trials previously investigated the clinical outcomes of diverse adjuvant therapy regimens. However, the optimal multidisciplinary treatment strategy is still controversial.

We hereby review the evidence on current and investigational adjuvant treatments for PDAC. Eligible studies were identified using PubMed electronic database for full manuscripts; abstracts and posters from conferences of international scientific societies were interrogated as well.

## 2. Chemotherapy

Historically, the first randomized trial evaluating adjuvant chemotherapy (CT) in resected PDAC was conducted back in the 1980s. Among 61 pts with resected pancreatic (*n* = 47) or ampullary cancer, post-operatory AMF (5-fluorouracil (5FU), doxorubicin, mitomycin C) regimen increased overall survival (OS) to 23 months from 11 months observed in the arm that did not receive adjuvant CT (p 0.04). However, this benefit was short-lived, and as early as at two years after randomization no difference in OS could be detected between arms (*p* 0.10). Even though the cure rate was not improved, this trial provided the proof of concept that adjuvant CT was able to impact on the disease course, and specifically by delaying the occurrence of disease relapse [9].

As the first adequately powered randomized trial evaluating adjuvant therapy in PDAC, the European Study Group for Pancreatic Cancer (ESPAC)-1 trial is considered a landmark study. It was characterized by a two-by-two factorial design, according to which each patient randomly received 5FU-based chemoradiation therapy (CRT) consisting of 20 Gy administered in 10 daily fractions, 6-month bolus 5FU-based CT, neither treatment, or both treatments administered sequentially. 289 pts were allocated to the treatment arms. OS was 20.1 months (confidence interval (CI)95% 16.5–22.7) among the 147 pts that received CT and 15.5 months (CI95% 13.0–17.7) among the 142 pts who did not (hazard ratio (HR) 0.71, CI95% 0.55–0.92; *p* 0.009); 5-y OS rates were 21% and 8%, respectively. The beneficial role of CT was independent of CRT receipt. Median (5-y rate) OS was 16.9 months (11%) among pts subject to observation, 13.9 months (7%) among pts treated with CRT, 21.6 (29%) with CT, and 19.9 months (13%) with CT-CRT [10]. The beneficial role of 5FU was later confirmed by a metanalysis of ESPAC-1 with other two trials of the ESPAC series (HR 0.70, CI95% 0.55–0.88) [11].

Gemcitabine (GEM), to this day the pivotal drug in the treatment of PDAC, was introduced into the adjuvant setting in the phase-3 Charité Onkologie (CONKO)-001 trial, in which 368 pts were randomly allocated to receive either GEM (1000 mg/m^2^ days 1,8,15/28 days for 6 cycles) or observation. Median disease-free survival (DFS) was doubled by GEM (13.4 months vs. 6.7 in the control arm *p* < 0.001); the beneficial role was consistent across all predefined strata, i.e., tumor stage, nodal status, and resection status. The benefit remained significant in the OS analysis (22.8 months vs. 20.2, HR 0.76 (CI95%, 0.61–0.95); *p* 0.01; 5-y OS 20.7% vs. 10.4%), although GEM could be received by relapsed pts in the control arm [12,13]. The CONKO-001 trial established a 6-month course of GEM as the standard of care for more than a decade. Its schedule for GEM administration would serve as model in the subsequent trials.

Similarly, GEM showed an advantage in terms of DFS even in the Asian population as suggested in the study by Ueno et al. [14] In a scenario where the beneficial role of adjuvant CT for surgically resected PDAC seemed to be established and both 5FU and GEM had proven effective in the adjuvant setting, the phase-3 ESPAC-3 (version 2) trial was designed to compare both regimens: 1088 pts were postoperatively randomized to either 5FU or GEM. No statistically significant difference was highlighted in both OS and DFS between treatment arms: OS was 23.0 months with 5FU (CI95% 21.1–25.0) vs. 23.6 with GEM (CI95% 21.4–26.4), and progression free survival (PFS) 14.1 months (CI95% 12.5–15.3) vs. 14.3 months (CI95% 13.5–15.6), respectively. However, serious adverse events were less prevalent in the GEM arm (7.5%) than in the 5FU arm (14%) (*p* < 0.001). Based on the better safety profile, GEM became the standard of care in the adjuvant treatment of surgically resected PDAC [15].

Adjuvant monotherapy could ensure long-term survival to only 16–21% pts, up until 2017, when the phase-3 ESPAC-4 trial provided the first significant move out of a years-long impasse. The doublet GEM plus capecitabine had previously shown better activity and efficacy than GEM alone in advanced PDAC, while maintaining an acceptable toxicity profile [16,17,18]. In the ESPAC-4 trial, 730 pts were randomly allocated to receive adjuvant GEM alone or in association with oral capecitabine (830 mg/m^2^ twice a day on days 1-21/28) for 6 cycles. After a median follow-up time of 43 months, OS was modestly prolonged by the addition of capecitabine to 28.0 months (CI95% 23.5–31.5) vs. 25.5 (CI95% 22.7–27.9) in the control arm, resulting in a clinically modest HR 0.82 (CI95% 0.68-0.98, *p* 0.032). As survival curves tended to a late separation, 5-y OS was improved (28.8% vs. 16.3%, respectively) but the OS difference at 2 years was less than 2%. The benefit from the combination treatment appeared confined to pts who underwent R0 resections (39.5 months vs. 27.9, *p* < 0.001), as opposed to those with R1 resections (23.7 vs. 23.0 months, p ns). Recent findings from a secondary analysis of this trial showed no significant differences between the time to recurrence and subsequent and OS between local and distant recurrence [19]. The study population of the ESPAC-4 trial was characterized by a worse prognostic profile than prior studies, highlighted by the high rate of pts with elevated post-operative blood carbohydrate antigen 19-9 (CA19.9) levels, tumor grade (G) 3 and lymph node (N) positivity, and reflected in the low survival observed in the control arm (5-y OS 16.3%), as compared to CONKO-001. This feature underscores the potential applicability of the ESPAC-4 findings to the real-life scenario. As for the safety profiles, the addition of capecitabine increased the incidence of G3-4 diarrhea (5% vs. 2%, *p* 0.008), neutropenia (38% vs. 24%, *p* < 0.001), and hand-foot syndrome (7% vs. 0%, *p* < 0.001), but the proportion of pts experiencing G3-4 adverse events was similar between arms [20]. As a result, the combination of GEM plus capecitabine for 6 months was established as the new standard of care following resection for PDAC, translating the improvements in tumor response and disease control obtained in advanced PDAC into the adjuvant landscape.

Shifting focus on experimental research conducted on Asian population, the phase-3 Japan Adjuvant Study Group of Pancreatic Cancer (JASPAC)-01 trial compared oral S-1 vs. GEM. 385 pts with an Eastern Cooperative Oncology Group performance status (ECOG PS) of 0 or 1 to receive a 6-month course of GEM or S-1 (40-60 mg on the basis of body surface area twice daily, 4 weeks on and 2 weeks off). OS was markedly prolonged in S-1 arm (46.5 vs. 25.5 months in control arm, HR 0.57, CI95% 0.44–0.72, *p* < 0.001). Moreover, S-1 exhibited a more favorable toxicity profile, with lower incidence of G3-4 leukopenia, neutropenia and transaminase elevation, and was associated with a higher quality of life than GEM. An intrinsic limitation of the study is that superiority of S-1 was not tested in the intention-to-treat but in the per-protocol population only [21]. Moreover, considering the reported differences existing in the pharmacokinetics and pharmacodynamics between Asian and Caucasian pts, it is difficult to assume that S-1 is universally superior to GEM, thus the results cannot be extended to the Western population.

The CONKO-005 trial, which found its scientific supportive background in the modest efficacy observed for the addition of erlotinib in the advanced disease [22], was the first phase-3 trial to investigate the adjuvant combination of a targeted agent with a classic cytostatic drug (i.e., GEM). This was a negative trial, in which a difference in DFS was not found (11.4 months in both arms, HR 0.94, CI95% 0.76–1.15, *p* ns) [23]. Ongoing analysis of tumor tissue samples of the CONKO-005 trial will hopefully identify a molecular subset of pts for whom Epidermal growth factor receptor (EGFR) inhibition provides maximum benefit. Similarly, the addition of sorafenib did not improve survival in R1-resected pts (CONKO-006 trial) [24].

More recently, the phase-3 PRODIGE 24–ACCORD 24-CCTG PA 6 trial investigated adjuvant mFOLFIRINOX, once again a regimen adopted from the advanced setting. In this trial 493 pts were randomly allocated to receive either a 6-month course of biweekly modified FOLFIRINOX (oxaliplatin 85 mg/m^2^, leucovorin 400 mg/m^2^, irinotecan 150 mg/m^2^ after an initial dose of 180 mg/m^2^, 5FU 2400 mg/m^2^ over 46 h), or GEM. The primary endpoint DFS was significantly prolonged, by 8.8 months, in the mFOLFIRINOX arm with respect to GEM (21.6 vs. 12.8 months, respectively; stratified HR 0.58, CI95% 0.46–0.73, *p* < 0.001). Remarkably, the DFS benefit with mFOLFIRINOX was significant in most subgroups, including those with adverse prognostic factors (e.g., T3 or T4 tumor, positive lymph nodes, or R1 resection). Pts allocated to the experimental arm experienced OS of 54.4 months vs. 35.0 in control arm (HR 0.64, CI95% 0.48–0.86, *p* 0.003), at the price of increased toxicity: mFOLFIRINOX treatment was associated with significantly higher incidence of G3-4 diarrhea, γ-glutamyl transferase increased, paresthesia, sensory peripheral neuropathy, fatigue, nausea, emesis, abdominal pain, and mucositis. Furthermore, in this trial a World Health Organisation performance status of 0 or 1 was an eligibility criterion, thus the observed difference in efficacy might have been magnified by the patient selection. Importantly, while the trial enrolled pts up to 79 years of age, and subgroup analysis showed no difference of effect between pts younger and older than 65, pts with at least 70 years of age did not gain any benefit from mFOLFIRINOX over GEM [25]. With these precautions, mFOLFIRINOX conferred an undisputable benefit to pts radically resected for PDAC and is poised to be the new standard of adjuvant care, or at least a viable therapeutic option, in fit pts with negligible comorbidity background and good recovery from surgery (Table 1).

## 3. Chemoradiation Therapy

Around one third of curatively resected PDAC pts will experience a local relapse in the absence of distant metastases [10,19]. The rational of integrating radiotherapy (RT) into the adjuvant therapeutic algorithm should be aimed to reduce the rate of local recurrence, which is as deadly as the metastatic spread, as eloquently demonstrated in the aforementioned ESPAC-4 trial. In this study, in which only 4% of pts received post-progression CRT [20], local recurrence was not associated with better post-progression survival compared to distant relapse [19]. However, if and how to associate RT with the widely accepted adjuvant CT option has long been matter of debate.

The potential of adjuvant CRT to improve patient survival was first showed in the 1980s by the Gastrointestinal Tumor Study Group (GITSG) 9173 study [29], in which 43 pts with resected PDCA and negative resection margins were randomized to observation or split-course CRT using 5FU as a radiosensitizer. RT was delivered to a dose of 40 Gy in 2 Gy fractions with a 2-week break after the initial 20 Gy. Despite the suboptimal radiation dose, and the low statistical power owing to the premature accrual termination due to slow accrual, the analysis of 43 treated pts revealed a statistically significant increase in OS in the CRT arm.

The survival benefit of celiac axis infusion (intra-arterial mitoxantrone, 5FU, leucovorin, and cisplatinum) combined with RT (54 Gy) versus observation after pancreatic cancer resection was investigated in a randomized controlled trial by Morak et al. [30]. No significant OS benefit was seen, but treatment schedule resulted in a prolonged time to progression.

Subsequent studies achieved conflicting results. In the phase-3 European Organization for Research and Treatment of Cancer (EORTC) 40891 trial, 218 pts with pancreatic head or periampullary adenocarcinomas were randomly assigned to split-course 5FU-based CRT (40 Gy) or observation [31,32]. No difference was observed either in terms of local relapse or OS between the two study arms. However, in this study, in which PDAC pts accounted only for a half of the study population, 50% and 20% of pts in the experimental arm did not receive CT per protocol or the intended adjuvant treatment at all, respectively.

In more recent times, the ESPAC 1 trial was a phase-3 factorial trial that randomized 541 pts to observation or adjuvant treatment with CRT alone, CT alone, or CRT followed by CT. A deleterious effect on survival emerged for adjuvant CRT, with estimated 5-year OS rates halved by the treatment [10]. This was ascribed to the delay of administration of CT following CRT, reducing the potential to eradicate micrometastatic disease as a consequence. However, definitive conclusions were impeded by several important limitations, among which the different time to the start of treatment in the different arms and the inadequate RT dose.

A subsequent large, phase-3 study, the Radiation Therapy Oncology Group (RTOG) 9704 trial [33,34] compared the role of GEM versus 5FU in addition to adjuvant CRT, consisting of dose of 50.4 Gy with concurrent continuous infusion of 5FU. No difference in terms of DFS or OS between the two arms was observed. Nevertheless, in the subgroup of pancreatic head tumors (388 pts), a trend of OS in favor of GEM was observed (*p* 0.08). A subsequent analysis further underlined the role of RT, observing that pts who failed to attain full exposure to per-protocol CRT administration experienced worse OS [35].

A randomized phase-2 trial, the EORTC-40013-22012/Federation Francophone de Cancérologie Digestive (FFCD)-9203/Groupe Coopérateur Multidisciplinaire en Oncologie (GERCOR) study evaluated adding CRT to adjuvant CT versus CT alone. This study enrolled 90 pts and compared two cycles of adjuvant GEM followed by CRT with concurrent weekly GEM to four cycles of adjuvant GEM The bimodal strategy proved slightly more toxic, and appeared to decrease the rate of first local recurrence (11% vs. 24% in the control arm), but no differences in DFS or OS were observed [36] (Table 2).

A network meta-analysis (2013) compared efficacy and toxicity of the major adjuvant treatments (5FU, GEM, CRT, CRT and 5FU, CRT and gem). With the caveats concerning heterogeneity of the included studies, CRT plus 5FU or GEM did not seem to provide a survival advantage over CT, with an increase in toxicities; moreover, CRT alone appeared to confer no survival benefit compared to observation.

This work reinforced the suggestion against proposing adjuvant CRT to unselected pts. Specific categories of pts had to be identified that could truly benefit from the addition of CRT to adjuvant CT. Among the most recent retrospective and registry studies (Table 3), an analysis of data from 6165 pts treated with adjuvant CT (38%) or CRT (62%) from the US National Cancer Database [39]. Most cases were classified as pT3 (72%), pN1 (67%) and R0-resected (84%). The median dose of RT was 50.4 Gy, whereas no details were provided concerning the CT regimens employed; included cases had RT initiated before the third cycle of CT. In addition to a small OS improvement with CRT compared to CT in the overall population, this analysis importantly circumscribed this observed benefit to pts with pT3 (HR 0.89, CI95%, 0.83–0.96, *p* 0.003) or pN1 (HR 0.86, CI95%, 0.79-0.92, *p* < 0.001) disease. On the contrary, resection margin status did not predict CRT efficacy over CT, with small gains observed both in R0 (HR 0.90, CI95%, 0.84–0.97, *p* 0.005) and R1 resections (HR 0.84; CI95%, 0.72–0.98, *p* 0.030). Limitations of this study include the lack of data on the baseline value of Ca19.9, the CT agents employed, the irradiated volume, and the radiation dose to nearby organs. However, the merit of this retrospective study lies in the identification of markers of benefit from CRT.

More recently, the US National Cancer Database was subject to a second analysis [46], aimed to compare the clinical outcomes of CT followed by CRT vs. either CRT and then CT alone, in the specific population with stage I-II resected PDAC. RT dose should have been of at least 45 Gy. The combined strategy (CT followed by CRT) achieved significantly longer OS than CT alone (median gap 3.3 months) and CRT alone (median gap 2.6 months). Again, the observed advantage from the combined strategy over CT or CRT was driven by N1 disease (HR 0.75), with no apparent effect in N0 resected tumors.

## 4. Adjuvant Treatment: Metanalyses

The reliability and applicability of data concerning adjuvant therapy in PDAC is often negatively affected by inter-study heterogeneity, regarding study population, stratification for prognostic contributors, and treatment options (surgery, CT regimens, RT dose, and techniques). Insufficient sample size is a frequent limitation among the published studies. A direct comparison between different adjuvant strategies lacking, metanalytic efforts tried to overcome these limitations and clarify the sometimes-unequivocal results emerging from randomized trials.

The first assessment of the role of adjuvant CT and RT [47] collected data from five randomized trials [9,10,26,29,31], of which the ESPAC-1, including the outcomes of 261 pts outside the factorial design (ESPAC-1 plus), provided the bulk of the sample (550 pts, or 63% of the total). After exclusion of cases diagnosed as periampullary cancer, data from 875 pts were analyzed at a patient level, except those coming from the old GITSG trial.

OS reached 19 months with adjuvant CT (CI95%, 16.4–21.1) vs. 13.5 months (CI95%, 12.2–15.8) with surgery alone, with significant, moderate HRs ranging from 0.65 to 0.75 depending on the selected trials; 2-y and 5-y OS were 38% and 19% for pts who received CT, and 28% and 12% for those who did not, respectively. To define the benefit from adjuvant CRT, data from ESPAC-1 (2×2 and Plus) and EORTC trials were pooled resulting in marginal heterogeneity (*p* 0.05). CRT did not confer any OS advantage (HR 1.09, *p* 0.43), with similar median values (15.8 months vs. 15.2 without CRT) and 5-y rates (12% and 17%, respectively). Even the inclusion of GITSG pts, characterized by a higher rate of R1 resections, only resulted in increased heterogeneity (*p* 0.02) with still no observed beneficial effect of CRT (HR 1.02, *p* 0.81). In the subgroup analysis, a non-statistically significant trend for higher OS with CRT vs. CT was observed for G3 tumors and for R1 resections.

The effect of adjuvant 5FU-based CT or CRT was investigated with the aim of establishing the absolute size of the OS benefit [48]. Five randomized or non-randomized prospective trials (totaling 670 pts) were included in the analysis: GITSG, EORTC, ESPAC1 2×2, the Norwegian Pancreatic Cancer Trial (NPCT) [29] and the non-randomized USA experience reported by Yeo et al. [49]. Other series, containing metastatic pts, were excluded. A statistically significant difference in 2-year OS between pts who received adjuvant therapy and pts who received surgery alone had not been achieved in any of the included studies. In the metanalysis, no significant inter-study heterogeneity was detected (*p* 0.459), and adjuvant therapy was associated with a significant gain in 2-y OS of 12% (*p* 0.011), a beneficial effect that was independent of the addition of RT in the adjuvant setting.

Another metanalysis [50] included data from the CONKO-001 [13] and the trial by Kosuge et al. [28], besides the published results of ESPAC-1 and the trials by Bakkevold et al. [9], and Takada et al. [26]. A statistically significant 3-month long OS prolongation (CI95%, 0.3–5.7, *p* 0.03) was associate with adjuvant CT but the gain in 5-y OS was marginal and not significant (3.1%, p ns). A statistically significant 3-month long OS prolongation (CI95%, 0.3–5.7, *p* 0.03) was associated with adjuvant CT but the gain in 5-y OS was marginal and not significant (3.1%, p ns).

The impact of type of radical resection on treatment efficacy was analyzed on four trials (*n* = 875) [51]: 68% of the sample had experienced a R0 resection, whereas 32% a R1; R1 resection was positively associated with nodal involvement (63% vs. 49% in the R0 group). Interestingly, resection margin involvement did not prove a significant prognosticator (HR 1.10, *p* 0.24); however, the beneficial effect of adjuvant CT on OS resulted greater in R0-resected pts (OS 20.8 months vs. 13.8 in pts treated with surgery alone), as compared to R1 resections (OS 15 months vs. 13.2). In contrast, CRT (*n* = 241) seemed to provide only a slight trend towards an OS advantage in R1-resected pts, emerging as a positive difference in median values of 3.5 months, as well as non-significant HR of 0.72 and 2-y and 5-y rates (30% vs. 19% with observation, and 18% vs. 8%, respectively). A trend towards a detriment on OS was observed in R0-resected pts (HR 1.19, CI95%, 0.95–1.49).

Xu et al. investigated the benefits of adjuvant CRT and neoadjuvant CRT in resectable pancreatic cancer. Seven studies were included in the comparison between CRT versus non-CRT and three studies were included in the comparison between neoadjuvant CRT versus post-operative CRT. In this meta-analysis CRT showed no significant effect on OS and PFS when compared to non-CRT, while neoadjuvant CRT showed no significant effect over post-operative adjuvant CRT [52].

A network metanalysis of nine trials [53] was able to perform a direct comparison among CT-only and combination strategies. The improvement in OS achieved by adjuvant CT with GEM or 5FU (HR 0.68 (CI95% 0.44–1.07) and HR 0.62 (CI95% 0.42–0.88), respectively) reached the full statistical significance after correction for nodal positivity, with conspicuous HRs of 0.59 and 0.65, respectively. The addition of CRT to any adjuvant CT regimen did not improve the efficacy with possibly worse outcomes than CT alone; moreover, CRT caused a relevant rise of toxicities, more so in association with GEM CT than with 5FU CT.

More recently, another network metanalysis [54] evaluated nine different regimens described in 13 trials. S-1 and GEM-capecitabine appeared the most effective agents for adjuvant CT, and again adding CRT to CT was confirmed unable to exert any significant improvement in OS.

## 5. Discussion

Despite the efforts cast in the recent past, even for the resected disease, the prognosis of PDAC remains dismal, with long-term survival rates at the lower end of the spectrum of the gastrointestinal malignancies [55,56]. In the wake of the benefit observed in the ESPAC-4 trial [20], GEM-capecitabine has surged as a valid therapeutic option; however the methodologic caveats and the subgroup analysis of this trial suggest caution in considering the ESPAC-4 regimen as the sole standard regimen in the adjuvant setting.

While S-1 has shown the potential to enter the therapeutic armamentarium for Asian pts [21], the results from the JASPAC-01 trial may not be generalized to Western population. However, the 5-y OS of 18%–44% achieved by nucleoside analogues, either in monotherapy or in combination, are clearly insufficient and call for the incorporation of active agents endowed with different cytotoxic mechanisms. The rationale relies both on exploiting synergic effects between different classes of antineoplastic agents [57], and on maximizing the chances of eradication of all the microscopic residues of a disease characterized by marked inter-tumoral heterogeneity [58]. The main road currently walked towards intensification is represented by the transposition of regimens which demonstrated meaningful efficacy in the advanced setting [25,59]. Indeed, a modified version of FOLFIRINOX markedly improved survival [60], whereas less neat results were divulged at American Society of Clinical Oncology(ASCO) Annual Meeting and European Society for Medical Oncology (ESMO) Congress 2019 for the Adjuvant Therapy for Patients With Resected Pancreatic Cancer (APACT) study, a phase-3 trial that evaluated the addition of nab-paclitaxel to GEM [61]. It is noteworthy that the experimental arms of both trials were compared against GEM alone, not with the doublet with capecitabine. Moreover, another significant limitation is represented by toxicity: the experimental regimens were associated with G3-4 adverse events in a larger share of pts (76–86%) than GEM monotherapy, even in selected populations that do not resemble the real-world typical patient who had undergone the invasive surgery required for PDAC radical removal. It is predictable that the mainstay of treatment of less-performing pts will not benefit from this kind of evidence and will remain based on GEM monotherapy or combined with capecitabine.

New evidence brought new issues, which add up to old, unresolved questions. Ongoing studies (Table 4) will hopefully provide an answer to part of them.

First, the identification of subgroups of pts in whom the efficacy of adjuvant CT is enhanced or diminished could be of help in the clinical practice. Unfortunately, easily retrievable prognostic factors, such as nodal involvement [62] and resection margins [63], failed to predict efficacy of adjuvant CT. With the caveats related to subgroup analysis (not pre-planned, reduced statistical power, p for interaction inconstantly reported), none of the five randomized clinical trials that reported one [10,20,21,60,61] could demonstrate a clear-cut difference of effect between arms according to the partition into these two subgroups. Molecular biology or immunohistochemistry can provide biomarkers that cut across the inconclusive clinical-pathological groupings. However, research in this field is still very immature, as only a few unvalidated predictive biomarkers of chemoresistance to adjuvant CT have been hypothesized so far [64,65].

Second, a major improvement in postsurgical survival could be represented by the incorporation of RT into the adjuvant setting. Unfortunately, in contrast to the CT approach, trials in this field are particularly scant. The few historical randomized trials were conducted during a large timespan, suffer with methodological flaws, and employed heterogeneous treatments. In particular, the employed RT dose was later recognized as suboptimal. Unsurprisingly, conflicting results and difficulties in data interpretation, the role of adjuvant RT is still controversial, even among international scientific societies. ASCO guidelines [66] recommend with a moderate strength to add adjuvant CRT after four to six months of systemic adjuvant chemotherapy for patients with lymph node-positive or R1 disease. In contrast, considering that survival benefit from adding CRT to adjuvant gemcitabine has not been definitely shown by strong evidence, ESMO guidelines do not recommend adjuvant CRT outside clinical trials [67]. Importantly, the recently released American Society for Radiation Oncology (ASTRO) guidelines lukewarmly endorse adjuvant CRT, for select high-risk patients defined by positive lymph nodes and surgical margins [68].

Against the hitherto presented backdrop, several issues on adjuvant CRT in PDAC still need adequate clarification. The evolution of RT techniques led to more effective radiation treatments, such as stereotactic body RT and intensity-modulated RT (IMRT), allowing more conformal dose delivery, and whose improved efficacy still need to be put properly on the test. A step further, the application to the post-operative setting of innovative techniques that are being pursued in other settings of PDAC care (NCT03536182, NCT03822936) could open new room for research.

On the other hand, the continuous improvement of adjuvant CT regimens -which in recent years increased to a great extent the chances of long-term survival after surgical resection [20,60]-could overshadow beneficial effects from CRT observed with less effective CT regimens. As a case in point, the ongoing phase-3 RTOG 0848 trial (NCT01013649) will provide useful data on optimal adjuvant CRT, in which an adequate dose is delivered also through IMRT, and to standardized, evidence-based target volumes. However, the employed adjuvant CT is still GEM monotherapy, so that dedicated trials will still be needed to elucidate the utility of and the efficiency in improving local control under these new conditions.

Considering that not all resected patients will benefit from adjuvant CRT, which on the other hand is not free from toxicities, more accurate negative and positive predictors of clinical benefit from CRT is a felt clinical need [45], which could find an answer in molecular biology and genetics. An ideal biomarker, or combination of biomarkers, should be able to indicate with high accuracy both the persistence of local disease, and its radiosensitivity.

Another line of research should further explore the addition of agents able to sensitize tumor cells to CT and CRT damage for systemic and local control, respectively. This approach has been initially investigated in locally advanced, inoperable PDAC with encouraging results [69].

Lastly, RT has showed an important effect on the immunosuppressive tumor microenvironment, which could be exploited to overcome the notorious resistance to immunotherapy typical of PDAC. Multiple studies currently are investigating PDAC vaccines (immune system activating pancreatic cancer vaccine (GVAX), Algenpantucel-L), in combination with CT or CT + CRT after surgical resection (NCT02451982, NCT01072981).

Third, a wider view of the treatment sequence will possibly improve the prognosis of primarily resectable PDAC. In particular, a few phase-2 randomized trials are evaluating the anticipation of CT to the preoperative setting. This approach should be able to ensure treatment administration to a higher proportion of pts than post-operative CT, the exclusion of early progressors from surgery, as well as decrease the rate of R1 resections. Interim results of the ongoing Neoadjuvant Plus Adjuvant or Only Adjuvant Nab-Paclitaxel Plus Gemcitabine for Resectable Pancreatic Cancer (NEONAX) trial (ASCO Annual Meeting 2019, abstract 4128 [70]) suggest the safety of the preoperative approach with GEM-nabpaclitaxel, whereas the combination of 5-Fluorouracil/Folinic Acid and Oxaliplatin (FOLFOX) and mFOLFIRINOX regimens are being evaluated in the PANACHE01-PRODIGE48 trial (NCT02959879). Indeed, preliminary efficacy outcomes from the phase-3 Prep-02/ Japanese Study Group of Adjuvant Therapy for Pancreatic Cancer (JSAP)-05 trial [71] favor the neoadjuvant approach with GEM-S-1 over upfront surgery in Asian pts, observing a 10-month, statistically significant difference in median OS.

## 6. Conclusions

After decades of slow progress, considerable advances have been recently made in the development of adjuvant strategies and protocols for resected PDAC, resulting in an unprecedentedly dynamic scenario, and sparkling new hope for the cure of this highly lethal, recalcitrant disease. However, at present survival rates remain unacceptably low, and dedicated research efforts are still urgently needed to improve the prognosis of these patients.

## Figures and Tables

**Table 1:** OS outcomes of the published randomized, controlled trials of adjuvant CT in PDAC.

Trial, Year of Publication; n; Geographical Region	Treatment Arms	Study Population		OS			Subgroups	
		N0	R0	Median (mts)	HR, *p*	5-Year Rate (Actual or Estimated)	Nodal Involvement	Resection Margins
Bakkevold et al., 1993 [9]; 61 (77% PDAC); Western	Dox, MMC, 5FU (6 cycles) vs. Obs	na	na	23 vs. 11	*p* 0.02	4% vs. 8%, *p* 0.6	na	na
Takada et al., 2002 [26]; 436 (36% PDAC); Eastern	MMC, 5FU (2 cycles) vs. Obs	PDAC						
		20%	58%	na	na, *p* ns	11.5% vs. 18.0%	na	R0: *p* 0.45 R+: *p* 0.75
ESPAC-1 and exp, 2004 and 2001 [10,27]; 289 and 541; Western	CT (LV, 5FU; 6 cycles) vs. CRT (20 Gy/10 fr, 5FU) vs. CRT + CT vs. Obs	41% 47% (exp)	82% 81% (exp)	CT vs. no CT				
				20.1 vs. 15.5	0.71 (0.55–0.92), *p* 0.009	21% vs. 8%	p_het_ 0.50	R0: 20.7 vs. 15.3, HR 0.65 (0.52–0.83) R1: 11.0 vs. 10.3, *p* ns
Kosuge et al., 2006 [28]; 89; Eastern	Cis, 5FU (2 cycles) vs. Obs	24%	100%	12.5 vs. 15.8	*p* 0.94	26.4% vs. 14.9%	na	na
CONKO-001, 2007 [12,13]; 368; Western	Gem (6 cycles) vs. Obs	28%	83%	22.8 vs. 20.2	0.76 (0.61–0.95), *p* 0.01	20.7% vs. 10.4%	N0: 34.0 vs. 27.6; HR 0.63 (0.40–0.97), *p* 0.04 N+: 18.5 vs. 18.2; HR 0.81 (0.63–1.06), *p* 0.44	R0: 21.7 vs. 20.8, p 0.18; HR 0.76 (0.60–0.98) R1: 22.1 vs. 14.1, *p* 0.07; HR 0.66 (0.39–1.13)
JSAP-02, 2009 [14]; 118; Eastern	Gem (3 cycles) vs. Obs	31%	84%	22.3 vs. 18.4	0.77 (0.51–1.14), *p* 0.19	23.9% vs. 10.6%	N0: 32.0 vs. 28.4; HR 0.63 (0.29–1.97), *p* 0.24 N1: 17.1 vs. 17.3; HR 0.84 (0.53–1.34), *p* 0.44	R0: 26.8 vs. 19.1; HR 0.70 (0.45–1.09), *p* 0.11 R1: 18.3 vs. 17.6; HR 1.05 (0.41–2.72), *p* 0.92
ESPAC-3 v2, 2010 [15]; 1088; World	FA, 5FU (6 cycles) vs. Gem (6 cycles)	28%	65%	23.2 vs. 23.0	0.94 (0.81–108), *p* 0.39	15.9% vs. 17.5%, *p* 0.39	p_het_ 0.60	p_het_ 0.56
ESPAC-4, 2017 [20]; 732; Western	Gem, Cap (6 cycles) vs. Gem (6 cycles)	20%	40%	28.0 vs. 25.5	0.82 (0.68–0.98), *p* 0.032)	28.8% vs. 17.5%, *p* 0.032	na	R0: 39.5 vs. 27.9 R1: 23.7 vs. 23.0
JASPAC-01, 2016 [21]; 385; Eastern	Gem vs. S-1 (4 cycles)	37%	87%	25.5 vs. 46.5	0.57 (0.44–0.72), p_ni_ and *p* < 0.001	24.2% vs. 43.6%, *p* < 0.001	N0: HR 0.51 (0.32–0.80) N1: HR 0.56 (0.42–0.75)	R0: HR 0.56 (0.43–0.73) R1: HR 0.57 (0.30–1.08)
CONKO-005, 2017 [23]; 436; Eastern	Gem, Erl vs. Gem	35%	100%	24.5 vs. 26.5	na, *p* 0.61	23% vs. 20%	no difference	-
PRODIGE 24, 2018 [25]; 493; Western	mFOLFIRINOX (12 cycles) vs. Gem (6 cycles)	23%	57%	54.4 vs. 35.0	0.64 (0.48–0.86), *p* 0.003	na	N0: HR 0.89 (0.53–1.49) N1: HR 0.54 (0.42–0.69) p_het_ 0.10	R0: HR 0.72 (0.53–0.98) R1: HR 0.52 (0.37–0.72) p_het_ 0.15

Abbreviations: 5FU = 5-fluorouracil; Cap = capecitabine; Cis = cisplatin; CRT = chemoradiation therapy; CT = chemotherapy; Dox = doxorubicin; ERL = erlotinib; exp = expansion; FA = folinic acid; Gem = gemcitabine; Iri = irinotecan; LV = leucovorin; MMC = mitomycin C; mts = months; na = not available; Obs = observation; OS = overall survival; Oxa = oxaliplatin; PDAC = pancreatic ductal adenocarcinoma; p_het_ = p for heterogeneity; p_ni_ = p for non-inferiority; CONKO: Charité Onkologie; JSAP: Japanese Study Group of Adjuvant Therapy for Pancreatic Cancer; ESPAC: European Study Group for Pancreatic Cancer; JASPAC: Adjuvant Study Group of Pancreatic Cancer; mFOLFIRINOX: oxaliplatin, leucovorin, irinotecan, 5-fluorouracil); PRODIGE 24: Partenariat de Recherche en Oncologie Digestive; HR: hazard ratio.

**Table 2:** OS outcomes of the published randomized, controlled trials of adjuvant CRT in PDAC.

Trial, Year of Publication; n; Geographical Region	Treatment Arms	Study Population		OS			Subgroups	
		N0	R0	Median (mts)	HR, *p*	5-Year Rate (Actual or Estimated)	Nodal Involvement	Resection Margins
GITSG 9173, 1985 [29,37]; 43; Western	CRT (20 Gy x2, 5FU) + 5FU vs. Obs	na	na	21.0 vs. 10.9	*p* 0.03	18% vs. 5%	na	na
EORTC 40891, 1999 [31,32]; 218 (PDAC 55%); Western	CRT (40 Gy/20 fr, 5FU)	39%	77%	15.6 vs. 12.0	0.91 (0.68–1.23), *p* 0.54	25% vs. 22%	na	na
ESPAC-1 and exp, 2004 and 2001 [10,27]; 289 and 541; Western	CT (LV, 5FU; 6 cycles) vs. CRT (20 Gy/10 fr, 5FU) vs. CRT + CT vs. Obs	41% 47% (exp)	82% 81% (exp)	CRT vs. no CRT				
				15.9 vs. 17.9	1.28 (0.99–1.66), *p* 0.05	10% vs. 20%	p_het_ 0.85	R0: 15.9 vs. 16.9, p ns R1: 10.9 vs. 12.1, p ns
RTOG 9704, 2008 [33,34]; 451; Western	CRT (50.4 Gy/28 fr, 5FU) + Gem (1 + 3 cycles) vs. CRT + 5FU (1 + 2 cycles)	34%	42%	Pancreatic head				
				20.5 vs. 17.1	0.84 (0.67–1.05), *p* 0.12	22% vs. 18%	na	na
Morak et al., 2008 [30]; 120 (PDAC 52%); Western	RT (54 Gy/30 fr) + i.a. Mit, FA, 5FU, Cis (1 + 5 cycles) vs. Obs	47%	73%	na	*p* 0.66	na	na	na
EORTC-40013-22012/FFCD-9203/GERCOR, 2010 [36]; 90; Western	Gem (2 cycles) + CRT (50.4 Gy/28 fr, Gem) vs. Gem (4 cycles)	30%	100%	24.3 vs. 24.4	na	na	na	na
CapRI, 2012 [38]; 110; Western	CRT (50.4 Gy/28 fr, 5FU, Cis, IFNα-2b) + 5FU (2 cycles) vs. FA, 5FU (6 cycles)	21%	61%	26.5 vs. 28.5	1.04 (0.66–1.53), *p* 0.99	na	na	na

Abbreviations: 5FU = 5-fluorouracil; Cis = cisplatin; CRT = chemoradiation therapy; CT, chemotherapy; FA = folinic acid; fr = fractions; Gem = gemcitabine; i.a. = intra-arterial; IFN, interferon; LV = leucovorin; Mit = mitoxantrone; mts = months; na = not available; Obs = observation; OS = overall survival; PDAC = pancreatic ductal adenocarcinoma; p_het_ = p for heterogeneity; GITSG: Gastrointestinal Tumor Study Group; EORTC: European Organization for Research and Treatment of Cancer; RTOG: Radiation Therapy Oncology Group; FFCD: Federation Francophone de Cancérologie Digestive; GERCOR: Groupe Coopérateur Multidisciplinaire en Oncologie.

**Table 3:** Recent studies comparing clinical outcomes of adjuvant chemoradiation therapy vs. other strategies in resected pancreatic cancer.

Author	Year	Clinical Study Design	N of Patients	Study Groups	RT Dose	RT Technique	Drugs	Key Results
Morganti et al. [40]	2014	retrospective	955	3: RT, CT, or CRT	45-60 Gy	na	fluoropyrimidines	OS: CRT 39.9 mts CT 27.8 mts RT 24.8 mts (*p* > 0.001)
Parikh et al. [41]	2015	retrospective	1130	3: surgery, CT, CRT	50 Gy	na	gemcitabine	OS: CT vs. surgery HR 0.71 (0.57–0.89) CRT vs. surgery HR 0.84 (0.69–1.02)
Rutter et al. [39]	2015	retrospective	6165	2: CT or CRT	50 Gy	na	na	CRT vs. CT OS: 23.3 vs. 20 mts 5-y OS: 19.6% vs. 16.5% (*p* 0.001)
Osipov et al. [42]	2017	retrospective	102	2: CT or CRT	na	na	na	>2 mm resection margin vs. <2 mm: Local RFS: HR 0.20 (0.05–0.88) OS: HR 0.31 (0.14–0.74)
Kanji et al. [43]	2018	retrospective	102	2: CT or CT-CRT	50 Gy	na	gemcitabine and taxanes, then fluoropyrimidines	CCRT vs. CT OS: HR 0.08 (*p* < 0.001) DFS: HR 0.23 (*p* 0.001)
Hsieh et al. [44]	2018	prospective	588	3: CCRT, CRT, CT	50 Gy	IMRT	gemcitabine	OS: CCRT vs. CT HR 0.40 (0.31–0.50) CRT vs. CT HR 0.31 (0.24–0.40) CRT vs. CCRT (*p* 0.014)
Xu et al. [45]	2018	retrospective	804	3: surgery alone, CT, CRT	50 Gy	IMRT	gemcitabine	CRT vs. no CRT: OS 23.7 vs. 17.0 mts *p* < 0.001 RFS 12.2 vs. 8.5 mts *p* < 0.001
Ma et al. [46]	2019	retrospective	5667	3: CCRT, CRT, CT	>45 Gy	na	5FU or gemcitabine	OS: CCRT vs. CT 23.3 vs. 20.0 CCRT vs. CRT 23.4 vs. 20.8 (*p* > 0.001)

Abbreviations: 5FU = 5-fluorouracil; CT = chemotherapy; CCRT = chemotherapy followed by concurrent chemoradiation therapy (CRT); DFS = disease-free survival; mts = months; OS = overall survival; na =, not available; RFS = relapse-free survival; RT = radiation therapy; IMRT = intensity-modulated radiation therapy.

**Table 4:** Pending results from randomized trials regarding either adjuvant or combined neoadjuvant and adjuvant treatments in resectable pancreatic cancer, as listed on www.clinicaltrials.gov, www.clinicaltrialsregister.eu and www.umin.ac.jp by September 2019.

Identifiers	Phase	Planned Accrual	Interventions	Primary Endpoint	Estimated Study Completion Date—Status
GIP-2 NCT02355119	III	310	Gem vs. FOLFOXIRI	DFS	Dec, 2018—status unknown
IMPRESS NCT01072981	III	722	Gem ± 5FU-based CRT vs. Gem ± CRT + Alg	OS	May, 2016—anticipated negative results
NCT02005419	IIR	300	Gem vs. Gem + Met	1-y RFS	Dec, 2017
NEPAFOX NCT02172976	II/III	40	Perioperative FOLFIRINOX vs. adjuvant Gem	OS	Jul, 2019
RTOG 0848 NCT01013649	II/III	545	Combination CT/Gem vs. Erl + Gem, followed by previous CT ± 5FU/Cap-based CRT	OS	Aug, 2020—partial preliminary results
HEAT NCT01077427	III	336	Gem + Cis + regional hyperthermia vs. Gem + Cap	DFS	Mar, 2021
SWOG S1505 NCT02562716	IIR	112	Perioperative FOLFIRINOX vs. perioperative nabP + Gem	OS	Oct, 2021
NCT02451982	I/IIR	75	Perioperative CTX + GVAX + Gem + RT vs. perioperative CTX + GVAX + Niv ± Ure + Gem + RT	IL-17A expression in resected tumors	Feb, 2023
NCT03959150	II/III	231	Gem + Cap followed by metronomic Cap vs. Gem + Cap	1-y DFS	Jun, 2023
NEONAX NCT02047513	IIR	166	Perioperative nabP + Gem vs. adjuvant nabP + Gem	DFS	Sep, 2024

Abbreviations: 5FU = fluorouracil; Alg = algenpantucel-L; Cap = capecitabine; Cis = cisplatin; CRT = chemoradiation therapy; CT = chemotherapy; CTX = cyclophosphamide; DFS = disease-free survival; Erl = erlotinib; Gem = gemcitabine; IL = interleukin; Met = metformin; nab = nab-paclitaxel; Niv = nivolumab; OS = overall survival; RFS = relapse-free survival; RT = radiotherapy; SBRT = stereotactic body radiation therapy; Ure = urelumab; y = year; GIP-2: Gruppo Italiano Pancreas-2; RTOG: Radiation Therapy Oncology Group; HEAT: Hyperthermia European Adjuvant Trial; SWOG: Southwest Oncology Group; NEONAX: Neoadjuvant Plus Adjuvant or Only Adjuvant Nab-Paclitaxel Plus Gemcitabine for Resectable Pancreatic Cancer; FOLFOXIRI: Combination of 5-Fluorouracil/Folinic Acid, Oxaliplatin and Irinotecan GVAX: immune system activating pancreatic cancer vaccine.
